# One Year of Online Education in COVID-19 Age, a Challenge for the Romanian Education System

**DOI:** 10.3390/ijerph18158129

**Published:** 2021-07-31

**Authors:** Eduard Edelhauser, Lucian Lupu-Dima

**Affiliations:** 1 Department of Management and Industrial Engineering, University of Petroșani, 332006 Petroșani, Romania; 2 Mining Engineering, Surveying and Construction Department, University of Petroșani, 332006 Petroșani, Romania; lucianlupu@upet.ro

**Keywords:** online education and emerging technologies, e-learning, COVID-19, socioeconomic impacts, educational challenges, Romanian education system

## Abstract

The study tried to analyze the implication of one year of online education in the Romanian education system. To achieve this goal, the authors of this study analyzed all the levels of education, primary education, lower secondary education, upper secondary education, and even the early childhood system, but also one of the smallest Romanian universities, considered representative for grade 1 universities representing 60% of the Romanian universities. The study is based on four online questionnaires for investigation, first with more than 2500 respondents from the primary and secondary Romanian education system, and the other three applied to more than 800 students and professors from the University of Petroșani. The investigation took place during 29 January 2021 and 11 February 2021. The authors had investigated the main feature of a standard online or a classical e-learning solution, such as the meeting solution or the video conference software, the collaborative work, such as homework or projects, and the testing method or the quizzes from both perspectives of the students and of the professors. The study results could influence the expected future hybrid educational system because these results were not covered in the previous literature but proved to be necessary for relevant knowledge strategies to be implemented in the new pandemic and also in the future context.

## 1. Introduction

After one year of the pandemic, countries are at different points in their COVID-19 infection rates, and worldwide, there are currently more than 1.2 billion children in 186 countries affected by school closures due to the pandemic. One year ago, in March 2020, governments began implementing measures to limit the spread of coronavirus, closing schools and moving to distance learning almost overnight. Approximately 150 countries fully closed their schools, approximately 10 countries partially closed them, and another 10 kept schools fully open. Globally, 214 million early childhood education to upper secondary education students in 23 countries missed at least three-quarters of classroom instruction time. Recent data show that over 90 percent of education ministries worldwide have implemented some form of remote learning approaches that involve radio, television, or the internet [1].

It is well known that for remote learning, a strong IT&C infrastructure is needed, but worldwide, significant differences exist, so some students without reliable internet access or technology struggle to participate in digital learning. This gap is seen across countries and between income brackets within countries. For example, whilst 95% of students in Switzerland, Norway, and Austria have a computer to use for their schoolwork, only 34% in Indonesia do [2].

Since the current COVID-19 situation began, there has been a significant increase in the uptake of e-learning solutions, including tools and methods such as video conferencing, virtual tutoring, digital libraries, and online learning software, causing a massive demand for online education connectivity. A mix-and-match of these tools with a variety of delivery methods, such as interactive e-learning courses, live and recorded lectures, and collaborative documents for group work, can work well to provide a comprehensive learning experience, but this also creates some difficulties for students and teachers. What has been made clear through this pandemic is the importance of disseminating knowledge across borders, companies, and all parts of society. If online learning technology can play a role here, it is incumbent upon all of us to explore its full future potential.

In the 2020–2021 scholastic year, the Romanian authorities announced different learning models for the new school year, based on each locality’s epidemiological situation. There were three scenarios: green—face-to-face classes, yellow—the hybrid model alternating online education or virtual learning and in-class or face-to-face learning, and red—the online education or at-home-only scenario. Most schools were reopened under the green scenario, while some use the other two learning models; however, despite this prognosis, for the first six months, the scholastic year was set to the red scenario equivalent to the online learning method after only two months. The Romanian educational system includes 2,900,000 students and over 200,000 teachers in the primary and secondary education levels.

On the other hand, a small Romanian university, University of Petroșani, was taken unprepared by the COVID-19 pandemic, and the implications in the field of education were huge. The management reaction in this area, the online education, and the e-learning platforms were not only very slow but also unsteady. The tools for online education were various because the decision to select a specific video conference software, a collaborative solution, or the testing methods remained the teacher’s option. This led to a not-homogenous method of education that was different from one teacher to other, and negatively affected the students.

The authors carried out this analysis in order to inform the Ministry about the perception of online education among teachers, and this approach was initially started only at the University of Petrosani level.

The research was carried out by analyzing the implication of one year of online education over the Romanian education system (RES) using the e-learning technologies. As main features of a standard online or classical e-learning solution used in RES, the authors considered the meeting solution or the video conference software, the collaborative work, such as homework or projects, and the testing method or the quizzes used by teachers and students or pupils.

In more than one year of forced online education caused by the pandemic, a huge number of studies have been developed, in different countries, in this field of the impact of online education on the education stakeholders. Some of these studies were studied by the authors in the literature review of this paper, and eight Romanian studies were also analyzed, but even so, the research has a high level of novelty for the Romanian education system developed on an online basis. The present study is probably the first Romanian research on the impact of online education on teachers and students from different levels of education at the same time.

Based on the fact that the present study is concerned with the Romanian primary and secondary education system (RPSES) and Romanian small universities, representing 88% of the Romanian pupils and students, the research is important for forcing the Romanian Ministry of Education to get involved in a strategic and planned manner, in order to prepare for the future Romanian hybrid education system.

The main goal of the study was to analyze the level of the implementation of the main feature of a standard online or a classical e-learning solution in the Romanian education system during the COVID-19 pandemic.

**Figure 1:**
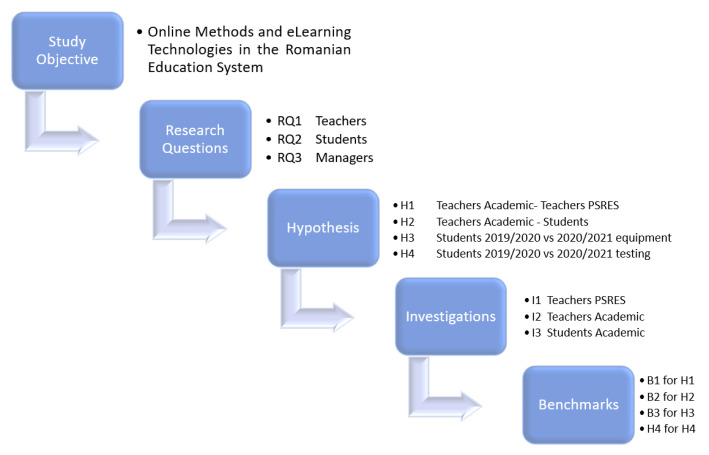
Conceptual research model.

**Figure 2:**
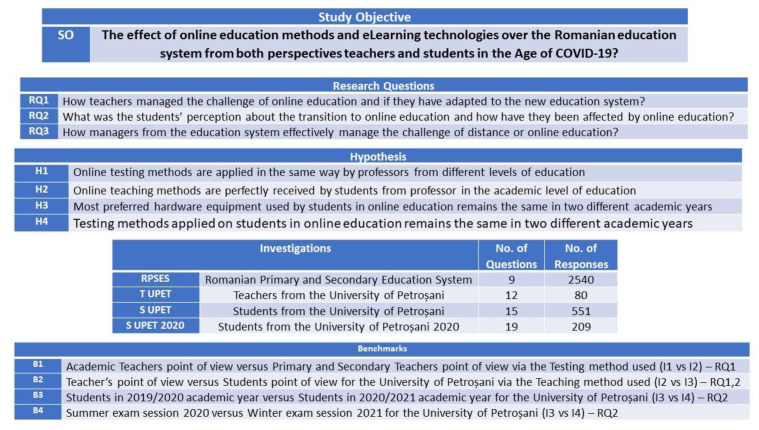
Detailed conceptual research model.

The study presented in this paper has a linear flow from study objective to research questions, research questions being supported by authors with hypothesis demonstration and benchmark tests, all of which are based on three investigations, as it can be seen in Figure 1 and Figure 2.

For supporting the three research questions, the authors set up four hypotheses, and for validating them, made four comparative analyses, the first of them between the perception of online education of the primary and secondary education teachers and the perception of academic teachers, the second one regarding the perspective on the online education from both parts, teachers and students, and the last two comparing the situation of online education in the University of Petroșani at the end of the academic year 2019/2020, and at the end of the first semester of the academic year 2020/2021.

## 2. Literature Review

The COVID-19 pandemic, perhaps one of the longest disruptive times, except times of wars, has irrevocably affected education and all related activities. Researchers around the world, whether they work in education or not, have studied the effects on all stakeholders involved in the education process. Many of them also tried to look to the future and to estimate how the new face of education will look.

A query of important databases such as the Web of Science Core Collection, Elsevier’s Scopus, The Directory of Open Access Journals, Springer, ScienceDirect, WILEY, and MDPI, using the keywords “e-learning, online education, COVID-19, virtual classroom, e-assessment, Romanian education system, ICT, advanced technologies” leads to almost 150 results. The evaluation of these works led the focus on more than 40 of them, in order to be analyzed in more detail. The analysis revealed three important issues that are correlated, and which are obviously caused by the COVID-19 pandemic. These issues have become major research themes and they have been transformed into the three research questions that support the theme of this paper.

The first major theme is related to the way in which teachers have managed to adapt to the new online education system. Teachers were the first to be affected by the forced transition to online education, because virtually no one was fully prepared to immediately migrate from face-to-face education to online education. The outbreak of the virus that caused the COVID-19 pandemic has found teachers, similar to anyone else, concerned about their own health, the health of their own family, and even of their students [3]. The enforcement of lockdown, in the conditions in which the activity had to be carried out, deepened the stress at the level of teachers, as a result of the need for the element of continuity in the educational process [3,4]. Besides this very important aspect, all teachers, depending on the conditions offered by the infrastructure of the educational institution in which they carry out their activities, were placed in front of an important set of choices:

- They had to quickly adapt their curriculum of the subjects they teach to the online teaching [5,6].
- They had to choose the most suitable online platform for them and for their students, in accordance with the preservation of the elements specific to the social needs of humanity, at an individual level [7].
- They had to choose the testing platform on which to run the student assessments and to transpose the tests according to the chosen platforms [8,9].

At the same time, the effective conducting of educational activities exclusively online has made teachers face a series of obstacles:

- Teachers have had to create special tools of human nature, in order to gain students’ trust as much as possible, since they were reluctant to open webcams during online courses [10].
- Teachers have had to diversify their online presence, appealing to social networks to be closer to students, to communicate better and more often with them [11,12].
- Teachers have had to increase the level of attractiveness of their courses, in order to capture students’ attention to online courses [10].
- Teachers have had to rapidly improve their digital skills to cope with the situation created by the COVID-19 pandemic [13].

All these challenges have shown a surprising aspect regarding teachers, namely a fairly high level of resistance to change [14]. This resistance, combined with the need to maintain a high level of quality of education, and even with the continuous increase of the quality of education [15], resulted, without doubt, in an online education process that proved to be more demanding and complex than the traditional one [16].

It was observed that teachers who had had digital skills coped better with the transition to online education, but even they were subjected to great pressure to perform their required teaching activities [17].

The second major theme is about how pupils and students were affected by the transition to online education and what their perception was. Naturally, along with the teachers, the students were also affected by the imposed transition to the online education.

The situation of the students is more diverse than that of the teachers, since they have a different structure from that of the teachers. Thus, there were differences between first-year students and the others, because for the first-year students, there is the stress of passing from one cycle of education to another. At the same time, the students had to use the technological resources offered by their family in order to be able to participate in the online courses, which led to an important technological handicap among them [18]. Because the students did not possess the level of maturity that would allow them to assess well the need for education, the situation created by the COVID-19 pandemic resulted in students’ avoidance of the educational process, from the participation to courses to the meeting of the teachers’ requests [14].

Overall, the students’ perception was rather favorable to the online education system:

- Students understood that the online education gives them a lot of freedom to connect with their teachers, colleagues, and to engage with their study materials in the comfort and flexibility of space and time [19].
- Students had the impression that the online education has increased their effectiveness and productivity [20,21].
- Students appreciated the translation of the content of the disciplines studied into e-content and found it very useful in the learning process [22,23].
- Students appreciated the use of multimedia materials as very useful and beneficial for their training [24].
- Students responded well in the correct abstraction of the content of the disciplines [25].
- Students were delighted that the teacher evaluation they complete has received new values regarding their adaptation to the use of digital and multimedia components in teaching [26].
- Students with poorer performance have benefited from the online educational system, which favors them by offering greater support than in the face-to-face system [27].
- Since the students developed constructivist activities of cognitive and social type, the imposed transition to the online system revealed the benefit of using massive Massive Open Online Courses (MOOC) courses [28].

There are also unfavorable aspects, not only from the point of view of the students’ perception, but also because of the collateral aspects. Thus, the students were reluctant to turn on the camera for reasons of privacy, which is why their ability to assimilate during online courses was reduced. It was necessary for the teachers to greatly increase the level of interactivity of the courses [10], and even so, the effects were not significantly better, because for students, too, the online education process is more demanding and more complex, and this leads to a loss of attention [16]. In addition, students’ expectations regarding the role of universities, governments, and authorities during the COVID-19 pandemic were not met, so they often felt left alone [29]. This is why the students perceived that the shift to the online education system has led to a decrease in employment opportunities [30]. On the same note, the students, especially the youngest ones, needed the full involvement of the parents in the educational process carried out online, so that they would be able to participate, to do their homework and the tasks they received from their teachers [31].

Students and pupils are beneficiaries of the educational system, which helps them to complete themselves as future adults. The transition from one education system to another, from the face-to-face system to the online system, cannot occur without the overall effort of all involved parties. For the acceptance of the online education to be good, indeed, it must be supported by other elements, related to the individual behavioral intention [32].

The third major theme is about how managers in the educational system have succeeded to manage specific activities in the context of the imposed transition to the online educational system. The first impression is that the success is weaker and insufficient, because not only teachers, but also pupils and students, carried, to a large extent, the weight of the online education period during the COVID-19 pandemic. During this period, no mechanisms were created to support the publication of scientific or didactic literature, so that the latter could be used to increase the quality of the educational system [33]. Expectations were created among the students regarding the actions of the managers from the educational system and even from that of the authorities; expectations that were not met [29]. Disregarding the lack of access to infrastructure for pupils and students led to inequities, because some of them could not benefit from education as did those with access to technology [34]. The COVID-19 pandemic period revealed a managerial crisis caused by the lack of correlation between management and the system itself, which showed that there was insufficient knowledge of the problems of the educational system at the level of management structures [35].

This has led to situations in which the negative effects of the online education were ignored [36].

However, there are favorable elements, in the sense of using the experience gained by the education management in this period affected by the COVID-19 pandemic, for the post-pandemic period. Management in education was surprised by, and unprepared for, the COVID-19 pandemic and by the enforcements caused by it, which is why the concept of an academic continuity plan appeared, as a transposition of the business continuity plan into the educational system. The implementation of such a system could facilitate the management of many potentially unforeseen situations [37]. In addition, only integrated actions can ensure an increase in performance in education, especially in the face of serious disruptive events, such as pandemics [32,38,39]. Thus, it is very important that the experience gained from the forced transition to the online be used as a source of improvement of the educational process [40,41]. In this regard, it is necessary for models to be followed for the migration to online education and also for the continuous training of the teaching staff to be supported and developed for a better adjustment to any form of development of the educational process, for creation of educational content, and for the increase of digital skills of teachers [42,43,44,45,46,47].

## 3. Methodology

**Figure 3:**
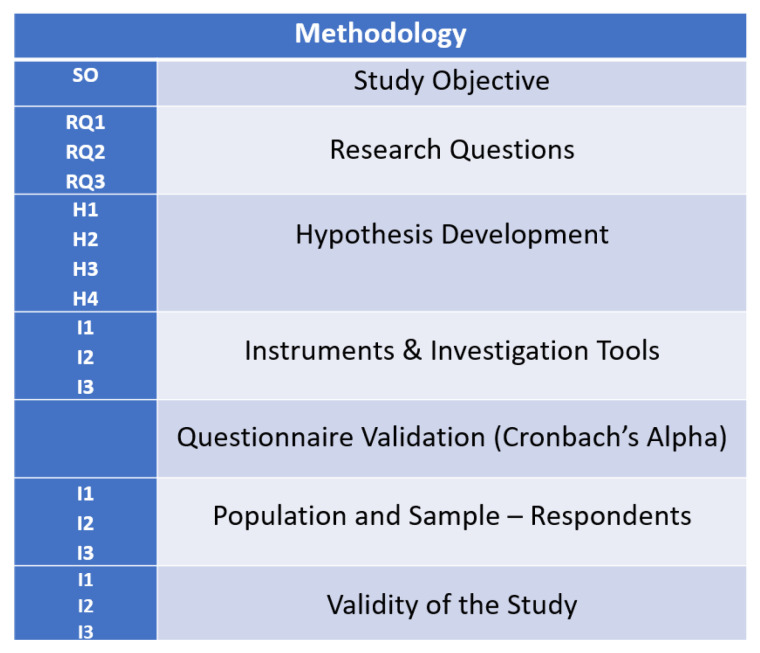
Research design structure.

The authors decided to carry out research based on online questionnaire investigations for analyzing the implications of one year of online education in the Romanian education system, as it can be seen in Figure 3.

It is obvious that adjusting teacher methods to the new reality or the perceptions of the students during the pandemic, or for the future hybrid education system, could be the result of research. But such research could be developed, in the authors’ opinion, only by the Romanian Ministry of Education, and our study results could represent a pilot test or they could offer the primary data for future research.

### 3.1. Study Objectives and Research Questions

The objective of the study set by the authors, after one year of online education, was to respond to an important question for the future of the education system: **What was the effect of online education methods and e-learning technologies on the Romanian education system from both perspectives of teachers and students in the age of COVID-19?**

This main objective of the study led the authors to the problem of answering three questions, which are, in fact, derivate objectives of the study.

Research Question 1 (RQ1): How teachers managed the challenge of online education and if they have adapted to the new education system?

Research Question 2 (RQ2): What was the students’ perception about the transition to online education and how have they been affected by online education?

Research Question 3 (RQ3): How managers from the education system effectively manage the challenge of distance or online education?

### 3.2. Hypothesis Development

Reviewing the vast literature dedicated to this year of global and forced online education, and also considering the objectives of this study, the following hypotheses were developed by the authors regarding online education teaching and testing methods, from different points of view of students and teachers, and also for two different academic years. The objective of setting this hypothesis was to demonstrate the effect that the implementation of the online education in the Romanian education system would have over the students.

**Hypothesis** **1** (H1)**.** *Online testing methods are applied in the same way by professors from different levels of education*.

**Hypothesis** **2** (H2)**.** *Online teaching methods are perfectly received by students from professors in the academic level of education*.

**Hypothesis** **3** (H3)**.** *The most preferred hardware equipment used by students in online education remains the same in two different academic years*.

**Hypothesis** **4** (H4)**.** *The testing methods applied on students in online education remains the same in two different academic years*.

These hypotheses are the result of the prestudies performed by the research team. The results were based on educational experiences that were not covered in the literature but proved to be necessary for relevant knowledge strategies to be implemented in the new pandemic context. In addition, the authors conducted similar research one year ago in May 2020 [48], based on a similar survey applied to 200 students of the University of Petrosani. In addition, during December 2020, the authors conducted a pilot study applied to 125 teachers from the Romanian Primary and Secondary Education System (RPSES).

### 3.3. Instruments and Investigation Tools

The online questionnaire was the investigation tool selected for this study, considering that an online survey is the most significant and reliable method for conducting this study. The collected data were processed by elaborating the structure of the data matrix and encoding the answers of the questionnaire applied. In addition, all the results of the questionnaires were processed using the IBM SPSS Statistics software version 23 (Armonk, NY, USA), where the variables were also verified.

#### 3.3.1. Investigation 1—Romanian Primary and Secondary Education System

The first investigation was based on an online questionnaire investigation, composed of 10 questions, five of them dedicated to the online education methods used, and three to the impact on the students, and the respondents answered in a standard time of 4 min per interview. The investigation took place during 29 January 2021 and 11 February 2021.

#### 3.3.2. Investigation 2—Teachers from the University of Petroșani

The second investigation was based also on an online questionnaire, dedicated to the University of Petroșani teachers. The questionnaire was composed of 12 questions: six dedicated to the online education methods used, and six to the impact on the students, and the authors received 80 answers out of 125 selected respondents, who answered in a standard time of 8 min per interview. In this case, the investigation took place during 29 January 2021 and 2 February 2021.

#### 3.3.3. Investigation 3—Students from the University of Petroșani

The third investigation was also based on a survey, composed of 15 questions: six dedicated to the online education methods used, and six to the impact on the students, and the authors received 551 answers out of 2153 selected respondents, who answered in a standard time of 9 min per interview, and the investigation took place during 29 January 2021 and 2 February 2021.

### 3.4. Questionnaire Validation

By using the three proposed questionnaires, the authors’ intention was to measure the level of the implementation of the main features of the standard online or classical e-learning solutions used in the Romanian education system during the COVID-19 pandemic.

The questionnaires were developed based on an exploratory and confirmatory pilot test study conducted in December 2020 for testing the content validity. The construct validity of the questionnaire was tested using the Pearson correlation matrix of major variables related to the online level, and data are presented in Tables 23 and 24. In addition, the fidelity and internal consistency of the questionnaire was tested using the Cronbach alpha for the multiple Likert questions.

After receiving data from the respondents, data were processed accordingly using descriptive statistics such as minimum, maximum, median, and standard deviation, used in the processing section of the questionnaires, as well as inferential statistics, such as Cronbach alpha coefficient to assess reliability, and Pearson r moment correlation and multiple linear regression (see also results from Tables 23 and 24). These statistical analysis tools were used to process the questionnaires applied to the target group. To transform the information from the questionnaires applied, the authors used the variables in SPSS: nominal, ordinal variables that are qualitative variables, and the range and ratio variables of type quantities, to assess the reliability, Cronbach coefficient was used; as indicated by Sekaran, a Cronbach alpha coefficient of 0.70 and higher is considered reliable and acceptable.

The authors assessed the reliability using the Cronbach alpha coefficient for the main variables, and the Cronbach alpha value of 0.850 indicates a high level of internal consistency for research with this specific sample of a standard online or a classical e-learning solution, and the correlation matrix shows the strength of the association between variables. Table 1 demonstrate that Cronbach alpha for all variables was well above the threshold of 0.70 recommended by Sekaran, and it can be deduced that the study meets the reliability hypothesis, and the reflective constructs have sufficient reliability.

### 3.5. Population and Sample Respondents

#### 3.5.1. Investigation 1—Romanian Primary and Secondary Education System

The authors began their study by contacting the 41 County School Inspectorates (ISJs) and asking for their help in applying the questionnaires to the Romanian teachers. Even if the survey was applied online and had only 10 questions for contacting the teachers from the primary and secondary education structure, the authors needed the ISJ’s help, and the ISJ’s provided help was the first remarkable aspect of the research. It is important to mention that Romania has eight development regions and more than 200,000 teachers [49].

From the 41 counties, the authors selected 18 counties with the ISJ’s support, and these counties were grouped in six out of eight development regions from Romania. As it can be seen in Table 2, the number of respondents, compared with that of the total number of teachers from the Romanian development region, represent 0.3% to 3.2% of the total.

#### 3.5.2. Investigation 2—Teachers from the University of Petroșani

The University of Petroșani has a reduced number of teaching staff, composed of only 142 teachers. The 80 respondents who answered the online questionnaire dedicated to academic teachers represent 64% of the 125 selected teachers by the authors, out of the total number of 142 teachers of the University of Petroșani. The 125 teachers were selected out of the 142, considering that the remaining 17 teachers have a small involvement in the teaching activities, such as the assistants. Even so, the number of respondents represent 56% of the total number of teachers of this small university, so the study is representative, and is probably the most representative study made for a small Romanian university in the online education period (Table 3).

#### 3.5.3. Investigation 3—Students from the University of Petroșani

This study was dedicated to the students of two faculties of the same university, the Science and the Mining Faculties. The total number of students of these two faculties is 2153, and it is also important to mention that the University of Petroșani has only three faculties, and the authors selected the Science and the Mining Faculties because their teaching activities involve students only from these two faculties, so the students could be easily contacted, and as a result their responses were in a representative number. The 551 answers represent 26% of the total number of the students, so the study is representative. The students of these two faculties who answered the questionnaire are undergraduate students in the bachelor’s programs (302), and graduates in the master’s degree programs (249). The average age of the undergraduates was 29 years, and the average age of the graduates was 35. In addition, from the total of 551 respondents, 283 students study engineering in the Faculty of Mining, and the other 246 students study economics and social science in the Faculty of Science, as it can be seen in Table 4.

From the statistical analysis based on the average age of the respondents, it can be observed that at the level of undergraduate studies, the Faculty of Mining has an average age of 10 years above the average age of a standard student. This could positively influence the acquisition of knowledge, with these adult students probably having previous practical experience, but they may also negatively adapt to the online system, as they do not belong to Generation Z, which is probably the first social generation to have grown up with access to the Internet and digital technology from a young age. For master’s degree students investigated, the age could positively influence the acquisition of information, and negatively influence the online adaptation, but on a lower level than it had affected the bachelor’s students.

### 3.6. Validity of the Study

The first investigation dedicated to the primary and secondary Romanian education system is representative by coverage area, in the authors’ opinion, and the second and the third investigations are relevant for the University of Petroșani, because the authors investigated 56% of the total number of teachers and also 26% of the total number of students of the two out of three faculties of the University. Obviously, this small university from Romania is representative only for the range of Romanian universities that have 3000 to 10,000 students, so-called grade 1 universities, representing 60% of the Romanian universities.

#### 3.6.1. Investigation 1—Romanian Primary and Secondary Education System

For question 3 and question 4, the authors unified ISCED 0 with ISCED 1 as primary level, because this type of education has particularities, and ISCED 2 with ISCED 3 as secondary level. According to the Ministry of Education, 2,918,275 students are enrolled in the 2020–2021 school year. Although they should all start school on 12 September 2020, the Minister of Education said at that time that 287,000 students do not have access to the internet and 237,000 students do not have devices to attend online school. The 524,000 students represent 18% of the whole Romanian student population, and this percentage is validated by the current study, because the investigated professors declare that the online education decreases the number of participants in classes, with 18% in the primary study cycle, and with 13% in the secondary cycle, as it can be seen in Table 5.

Represented in Table 5 is the level of the adaptability of the teachers to the online system. Both previous tables, Table 5 and Table 6, confirm through the collected data that the lower level of attendance was in the primary system, 82%, but on aggregate, the decrease of student attendance and the lack of adaptability of the teachers is in the range of 13 to 18% for the students (Table 5) and 19% for the teachers (Table 6). Questions 2, 3, and 4 confirm that this study is valid because these demonstrated percentages of decrease of participation to online classes are equal to those presented by the Ministry of Education on a national level, so the answers of the 2540 respondents are representative.

#### 3.6.2. Investigation 2—Teachers from the University of Petroșani

From Q1 and Q2 as it can be seen in Table 7 and Table 8, the authors can conclude that only 11% to 15% of the number of professors consider that online education conducted resulted in a decrease from both points of view of teachers and students, rated in the 18% limit declared by the Ministry officials, an observation that can support the conclusion that the study is valid for the academic level of education. Questions 2 to 6 confirm that this study, investigation 2, is valid because the demonstrated percentage of decrease of participation in online classes is equal to that presented by the Ministry of Education at a national level, so the survey respondents are representative at a national academic scale.

#### 3.6.3. Investigation 3—Students from the University of Petroșani

This investigation is also valid for the academic level of education because student participation in online classes was placed in the national range of participation, as it can be seen in Table 9.

The present research is a consequence of the research developed by the authors one year ago at the University of Petrosani after only two months of online education. The authors are involved also in training courses for the pre-university teaching staff, especially in the IT&C field, and these courses offered them the opportunity to investigate, in December 2020, a pilot sample of teachers from RPSES involved in online education. In this context, the authors have observed that the change represented by the transition from classical to online education for the Romanian education system is a fundamental one without discussion, while until March 2020, online and e-learning methods had a low usage in the small Romanian universities and in the RPSES; concerns existing only in universities that offered courses in distance education. This was the reason why even the study has two different and not totally homogeneous parts, as the reviewer observed, but with this similarity regarding the online education.

The authors investigated the University of Petrosani, a small university that could be considered representative not for the whole Romanian education system, but for the small Romanian universities. This assumption can be based on the fact that primary, secondary, and high schools, and small universities also, are similar in fields of online education, because, unfortunately, they started the massive implementation and use of e-learning only after 15 March 2020. In addition, primary, secondary, high schools, and small universities represent 88.80% of the pupils and students involved in the Romanian education system, while 327,471 students follow the courses of the top universities, the first 17 Romanian universities that have more than 10,000 students and had a developed online system for distance education, from the total of 2,926,168 pupils and students from Romania.

The Romanian National Council for the Financing of Higher Education statistics offer the total number of the Romanian students for the 2019/2020 academic year as 459,899. The same statistics show that from the total of the 49 Romanian State Universities, 32 are small universities having around 5000 students, and representing 30% of the Romanian students. On the other hand, these small universities are very important and are representative because most of them are comprehensive universities having the most important fundamental areas of study such as engineering, social science, and humanities. The University of Petrosani is a such small university, having 3565 students and as areas of study engineering and social science.

In the field of e-learning, all these small universities are similar, they have no previous distance learning, so they have had to adjust very quickly to the new conditions of education generated by the COVID-19 pandemic. In addition, these small universities could not afford to buy an e-learning platform produced by one of the major world players from this field. These small universities have adopted low-priced e-learning platforms such as LMS’s, developed on Moodle, or free collaborative educational platforms.

In conclusion, the research is applicable only to the RPSES and the grade one Romanian universities, small universities with less than 10,000 students, and not for the first top 17 Romanian universities.

## 4. Results

**Figure 4:**
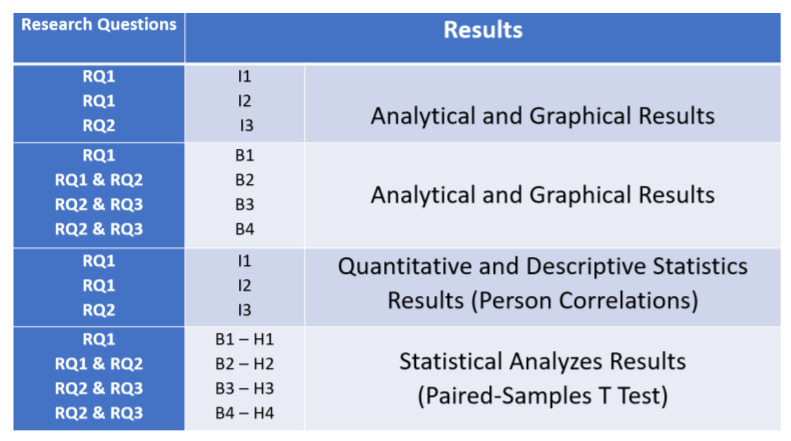
Results section structure.

The Results section of this study is structured on four subsections, all based on the three investigations and four benchmark tests for supporting the hypothesis. All the four subsections were developed for supporting the three research questions from the study, as it can be seen in Figure 4.

### 4.1. Analytical and Graphical Results

#### 4.1.1. Investigation 1—Romanian Primary and Secondary Education System (RPSES)

The investigation made in RPSES included different curricular areas, and teachers from these various educational areas were using different methods of teaching; some of them use visual materials, some are recording their lectures, while others film their demonstrations made on flipcharts and send them to the students. From the teaching methods, PowerPoint presentations or video meeting are preferred by more than 66% of the teachers as a teaching method, but even so, probably more than 40% of the used methods were only documents in a .pdf form sent by email, as it can be seen in Table 10.

One interesting observation is that teachers from primary education level use WhatsApp for meetings a little bit more, while teachers from secondary education level use more sophisticated methods for meetings, such as Microsoft and Google solutions, as it can be seen in Table 11.

As a collaborative platform in the Romanian primary and secondary education system, Google G Suite for Education is maybe 20 times more used than Microsoft Office 365 Academic or any other collaborative platform, , as it can be seen in Table 12.

In the online classes, the testing method has an important role similar to that of the video meeting, but using quizzes from collaborative platforms such as Google, Microsoft, or Class Marker brings novelty to classic testing systems, considering the time pressure, the objective method of testing, or the impersonal feature of a digital platform. Probably, that is why teachers from the primary and secondary education system prefer other methods of testing, such as homework or video meetings, as it can be seen in Table 13.

As a homework transmission method, WhatsApp is widely used in the primary and secondary education system, but because the great majority of the schools and high schools have implemented virtual classrooms, especially Google Classrooms, by setting institutional emails for students and pupils, the virtual classroom is also very much used, as it can be seen in Table 14.

Results from Table 10, Table 11, Table 12, Table 13 and Table 14 support the RQ1 and demonstrate that the main features of a standard online or a classical e-learning solution were used by teachers from the RPSES during the COVID-19 pandemic, and teachers have adapted to the new education system.

#### 4.1.2. Investigation 2—Teachers from the University of Petroșani

From the question related to the hardware devices used to run the online courses, with answers obtained by choosing the degree of use for each option (Q7), the authors observe that the most used hardware equipment for online courses was, for the University of Petroșani (UPET) teachers, obviously the laptops, with 82% of them preferring this versatile equipment. This trend is correlated with the greatest sale increase of the last 10 years, with 28% of the laptops during the COVID-19 pandemic, and also the sale decreases, with 26%, of the desktops in the same period. Smartphones are not the favorite equipment for teachers, in fact, this equipment is not suitable at all for teaching online, maybe just for attending online classes. In this context, it is also relevant that the market sales have dropped down in 2020 by 20% for smartphones.

From the question related to the method of communication used by teachers to keep in touch with students during the COVID-19 pandemic (Q8), with answers obtained by choosing the degree of use for each option, the authors observe that at the academic level, compared to primary and secondary education level, email is a very used and efficient tool for online communication. In fact, only the favorite meeting tool for the University of Petroșani members, Zoom, has a bigger reliance for teachers. It is also relevant that Facebook groups or other social media tools, used mostly by students and teachers before the pandemic for communication, have dramatically dropped down in their preference in the pandemic period.

From the question related to the teaching method used during the COVID-19 pandemic at UPET by teachers (Q10), the authors observe that in the COVID-19 age, the University of Petroșani teachers maintained their classical methods of teaching for the students, especially PowerPoint presentations, that could be very easily shared to the students via the share screen option of the meeting software used. In a small percent, 24%, for an academic institution, collaborative projects and eContent methods were also used.

Results from Q7–Q12 from Investigation 2 also support the RQ1 and demonstrate that the main features of a standard online or a classical e-learning solution were used by the teachers from University of Petrosani during the COVID-19 pandemic, and teachers have adapted to the new education system.

#### 4.1.3. Investigation 3—Students from the University of Petroșani

From question 3 and question 4 (What was the average number of students from undergraduate studies to a specialization or year of study participating in courses before the pandemic? and What was the average number of students from undergraduate studies to a specialization or year of study participating on courses during the pandemic?), the authors conclude that the number of bachelor’s students raised by 9% from a median number of 16.75 students per class to a median number of 18.17 students per class. Even if there is small increase in participants, this increase represents an advantage, especially for the students having their residence outside Hunedoara County, and especially outside Jiu Valley, where the University of Petrosani is situated.

From question 5 and question 6 (What was the average number of students from master studies to a specialization or year of study participating on courses before the pandemic? and What was the average number of students from master studies to a specialization or year of study participating on courses during the pandemic?), the authors conclude that the number of master’s program students attendance raised by 33% from a median number of 13.14 students per class to a median number of 17.47 students per class. This important increase of this attendance is also an advantage, especially for the students having their residence outside Hunedoara County, and especially outside Jiu Valley zone. The authors must mention that only 66 out of the 80 teachers conduct classes for the master’s program students.

Questions 7 to 10, related to the online education methods used in the COVID-19 period, were analyzed by the authors in benchmark 2, using also questions 7 to 11 from investigation 2 as a comparison.

Questions 11 and 12 were correlated, resulting in an unexpected result (question 11: What was the number of hours per day dedicated to your online courses during the COVID-19 pandemic? and question 12: What was the number of hours per day dedicated to conducting classical courses by you in the period before the COVID-19 pandemic?), demonstrating that the University of Petroșani is not prepared for online, and even if the classes are not compulsory in the Romanian academic system, the class drop-out rate was 8% in one year of online education. From the point of view of some of the teachers, the number of students had raised the class attendance, but the student responses demonstrate that their participation was lower. This could be caused by the lack of implication of professors in online education, or because they have incompletely adapted to this system, or by IT lower skills from both parts.

Even if only in the bachelor engineering classes there is a raise of participation (line 1 from Table 15), the median participation for the bachelor’s programs during the online system is equal with the previous one from the classical system. On the other hand, in the master’s program activities, the students feel a lack of interest from the professors or from this teaching method used by them during the pandemic crisis, and the attendance has dropped. In addition, this forced online teaching method imposed by the COVID-19 pandemic resulted in a low level of interaction between teachers and students, and the feedback from the students was almost absent, because only a face-to-face interaction could probably lead to a high level of student-teacher engagement. In many situations, teachers report, during formal discussions, dysfunctions concerning the following learning support activities: authentic communication and human relationships, and personalized support for students with special learning needs.

In the Romanian academic educational system, the semester curricula consist of five to seven study disciplines. Table 16 shows that 8% of the students support the idea that the number of online conducted classes was only three out of seven. Considering this, the investigation shows that there is a drop of 8% in the participation, using the results of question 11 and question 12, and this is probably especially because of the teacher’s noninvolvement, considering that in the academic education, technical difficulties are not relevant.

The investigation demonstrates also that 16% of the students attended only 50% of the video conference-conducted classes, representing only one to three courses out of seven, but this deficiency can be determined by teachers’ low level of IT skills, by technical difficulties such as safe internet connection, or the lack of responsibility of both sides.

Considering that the highest level of online teaching is represented by e-learning platforms, meaning virtual classes and online quizzes, the authors demonstrate that 38% of the students participate in classes conducted through an e-learning platform, in only one to three courses out of seven, representing 50% of the classes, and this could be also caused by a wide range of facts. Among the difficulties in carrying out distance teaching activities, teachers signal in interviews made by the authors, in order: the lack of tools for class management for feedback and for evaluation, technical difficulties: platforms errors, platforms not working, lack of pedagogical support for carrying out sufficiently effective learning activities and/or attractive to all students, lack of appropriate tools for teaching-learning assessment to their discipline, the lack of educational content (digital resources) in the field discipline, lack of a sufficiently performing computer, and also lack of time for understanding and proper use of digital tools and resources.

Results from Q3–Q6 and Q11–Q14 from investigation 3, and Table 15 and Table 16, support the RQ2 and demonstrate that the main features of a standard online or a classical e-learning solution were used by the students from University of Petrosani during the COVID-19 pandemic, and students have partially adapted to the new education system.

#### 4.1.4. Benchmark 1—Academic Teachers’ Point of View versus Primary and Secondary Teachers’ Point of View

The authors analyzed the difference in perception for teaching method used by academic teachers and primary and secondary teachers, and they observed small differences considering the different topics in the academic stage and those in the primary and secondary level. Therefore, it was obvious that video presentations were preferred by pupils, while eContent and collaborative projects are mostly preferred by students.

The authors also analyzed the difference in perception for video meeting method used by academic teachers and primary and secondary teachers, and they observed that the meeting solutions were widely used both at academic level and primary and secondary level, so the only difference was that in the University of Petroșani, the large majority of the teachers chose the Zoom solution, while in the primary and secondary education system, the Google Meet solution was selected. It is also relevant that WhatsApp remains an option only for the primary education level.

**Figure 5:**
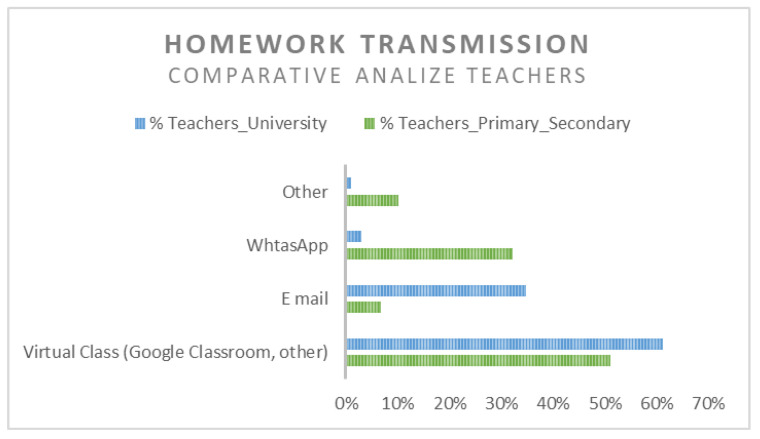
Different perception for homework transmission method used by academic teachers and primary and secondary teachers.

The authors then analyzed the difference in perception for testing methods used by academic teachers and primary and secondary teachers, and they observed that in the testing area of the online education, the academic teaching staff, as well as the primary and secondary education teaching staff, have chosen the Quizzes methods, using the forms from Google, Microsoft, Class Marker, or their own developed testing module, with small differences being visible between the two sample populations, as it can be seen in Figure 5.

The only relevant difference in the homework transmission method used by the students and pupils is that while email is an academic solution, WhatsApp can be considered a solution for the primary education system.

Benchmark 1 results demonstrate similarities and differences between the main features of a standard online or a classical e-learning solution in the teacher options of two different education systems related to RQ1.

#### 4.1.5. Benchmark 2—Teachers’ Point of View versus Students’ Point of View for the University of Petroșani

The most-used hardware equipment for online courses are, for the University of Petroșani students, obviously, the smartphones, but the laptops as well, with 87% of them preferring these two pieces of equipment, and for the University of Petroșani teachers, obviously the laptops, with 82% of them preferring this versatile equipment. Smartphones are the favorite equipment for the students, but these are suitable only for attending classes online, and not for teaching online classes, and that is why smartphones are not the favorite equipment for the teachers. In this context, it is also relevant that students have difficulties when the teachers ask them to present a paper via share screen from a smartphone. The extensive use of smartphones could also imply that students pay less attention to the educational process and are less interested in the educational process while they are online, because phones offer a high level of mobility for the students.

In the academic field, despite the primary and secondary education, the email is a very used and efficient tool for communication online. In fact, the only favorite meeting tool for the University of Petroșani members, Zoom, has a similar reliance for students.

**Figure 6:**
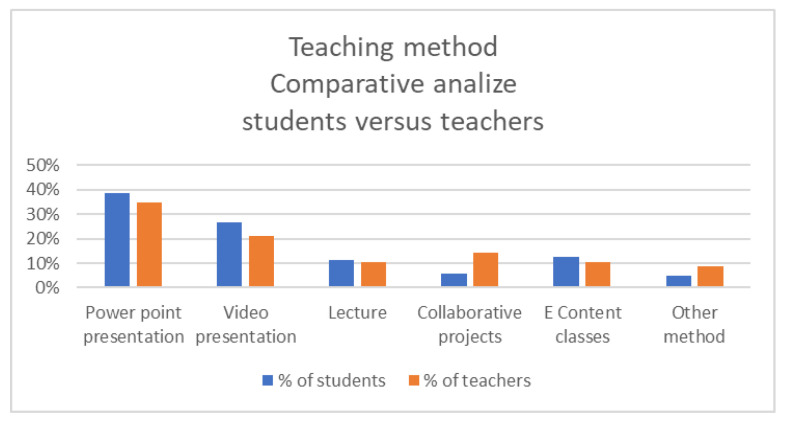
Different perception for teaching method used, teachers versus students.

In the COVID-19 age, the University of Petroșani students receive courses from their teachers in the classical methods of teaching, especially PowerPoint presentations that could very easily be shared to the students via the share screen option of the meeting software used. In a very small percentage, 18%, for an academic institution teaching process, collaborative projects and eContent methods are also used from the student perspective, even if teachers declare that these methods are used in a moderate level, 24%, from the professor’s perspective, as it can be seen in Figure 6.

**Figure 7:**
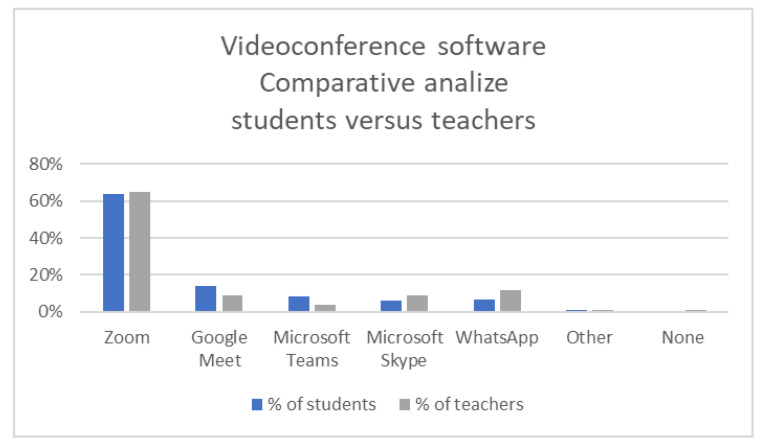
Different perception for meeting tools used, teachers versus students.

Even if an online semester has already passed, the University of Petroșani management board did not select a unique software for meeting conferences at the beginning of the 2020/2021 academic year, so students were forced to use different meeting solutions for online classes, and this affected them in an unpleasant way, with their attention being shifted from the course to online technical issues for using different meeting applications. Even if more than 64% of them attend online classes conducted by Zoom meeting solution, the other meeting solutions were also used, as can be seen in Figure 7.

**Figure 8:**
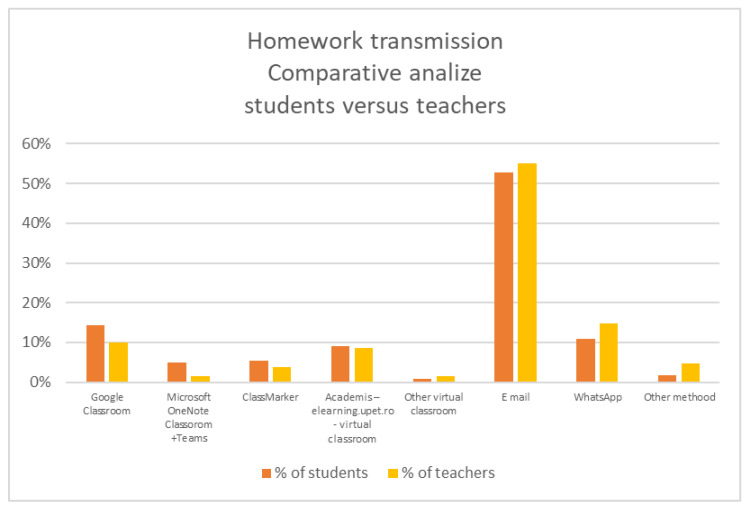
Different perception for homework transmission used, teachers versus students.

**Figure 9:**
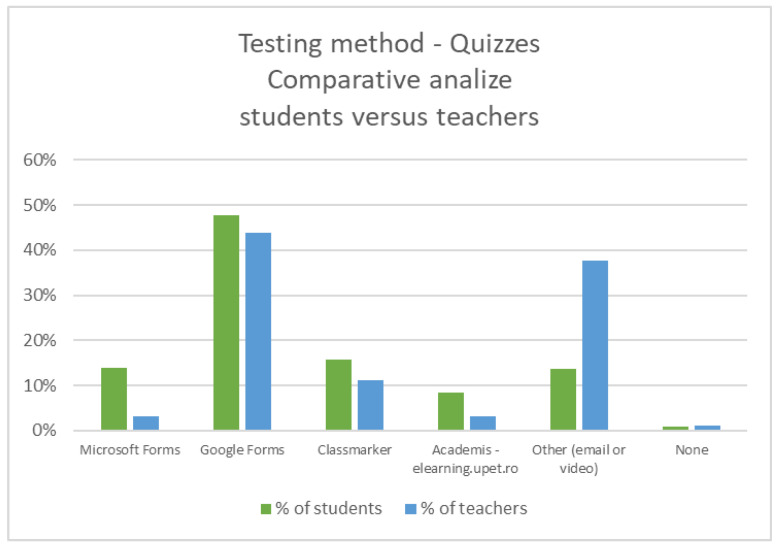
Different perception for homework transmission method used, teachers versus students.

For the fall semester of the 2020/2021 academic year, the University of Petroșani implemented a poorly developed e-learning platform called Academis, developed without a meeting solution, with a rudimentary Quizzes module and without a self-recording presence module, so students were restricted for homework or projects transmission to use mainly email, at 53%. The new implemented platform was used only by 9% of the university students, and even WhatsApp represents a more used solution for them, as it can be seen in Figure 8 and Figure 9.

Quizzes were used as a testing method for 86% of the students, being perceived as the most widely used method of examination, with different test forms such as Google, Class Marker, or Microsoft, but even so, 13% of the students mention as an evaluation method the homework or project transmission via email, as it can be seen in .

Benchmark 2 results demonstrate similarities and differences between the perceptions of the main feature of a standard online or a classical e-learning solution in the teachers’ and students’ options, and are related to RQ1 and RQ2.

#### 4.1.6. Benchmark 3—Students in the 2019/2020 Academic Year versus Students in the 2020/2021 Academic Year for the University of Petroșani

This benchmark is analyzed in the Statistical Results section.

#### 4.1.7. Benchmark 4—Summer Exam Session 2020 versus Winter Exam Session 2021 for the University of Petroșani

Quizzes, generated in Microsoft, Google, or other collaborative software, remain a testing method stated by the professor for the great majority of the students, as it was stated as the most widely used method of examination, with different test forms such as Google, Class Marker, or Microsoft, in the official Deans records situation. For the students of the Science Faculty, around 50% of the exams were set in a Quizzes mode, while for the Faculty of Mining, there was a 24% decrease but also a 16% increase for the mixed method of examination video conference and Quizzes. The most relevant difference after almost a year of online education is the disappearance of WhatsApp exams, and also the reduced number of video conference exams, especially in the engineering field (Table 17).

To analyze the adaptability, effect, and efficiency of this particular year in the field of education, the authors investigated over 3400 respondents who implemented or received online education. First, in a survey conducted at the end of April 2020, they identified how online education was perceived after only one and a half months of pandemic, from the perspective of over 200 students of the University of Petroșani, and the results were presented in another paper [48]. Then, they compared the perspectives of those 200 students with the perspectives of another 550 students, representing 25% of the two investigated faculties of the University of Petroșani, but this time at the end of January 2021, after 10 months of online education. Because there is a big difference between the beneficiary and the provider, they analyzed online education from the teachers’ perspective, and over 50% of UPET teachers responded to the challenge; in fact, 80 teachers out of 145, and because the University of Petroșani, through its 4000 students, is one of the smallest universities in Romania, for obtaining a global perspective, data from the pre-university system, or primary and secondary education system where needed. Therefore, they investigated over 2500 teachers, distributed in representative shares of the pre-university system, respectively 39% of the primary and early childhood education level, 36% of the gymnasium cycle (lower secondary education), and 25% of the high school cycle (upper secondary education), with a national coverage of six out of the eight development regions of Romania [49].

Benchmark 4 results demonstrate similarities and differences between the perceptions of the one main feature of a standard online or a classical e-learning solution, the testing one, in the students’ opinion in two different academic years, and is related to RQ2.

### 4.2. Quantitative and Descriptive Statistics Results

#### 4.2.1. Investigation 1—Romanian Primary and Secondary Education System

For the first investigation, the respondents were not homogeneous, coming from 18 different Romanian counties, and that is why data were very diverse.

Table 18 presents the average score for the items of attendance before and during COVID-19, and demonstrates that teachers from primary and secondary education system detect a drop, in terms of the student number involved in teaching activity.

Questioning a large number of respondents, the online teaching environment was very diverse, but despite the large number of teaching methods used (6), video conference solutions (5), communication (4), and testing methods (4), the averages of this online parameter remain between 1.04 and 2.42 (Table 19).

#### 4.2.2. Investigation 2—Teachers from the University of Petroșani

For the second investigation, based on the teachers’ response of a small university, it was considered important the perception of the teachers regarding the level they manage to reach in the online teaching process, and also the level of student attendance to the classes.

The level of teaching process was measured in a four-scale-levels representation, considering that 4 was “identical”, 3 was “slightly reduced from 80% to 99%”, 2 was “reduced from 50% to 79%”, and 1 was “very low below 50%”. In addition, the level of attendance was measured in a five-scale-levels representation, considering that 5 was “higher, from 110% to 150%”, 4 was “identical”, 3 was “slightly reduced from 80% to 99%”, 2 was “reduced from 50% to 79%”, and 1 was “very low below 50%” (Table 20).

#### 4.2.3. Investigation 3—Students from the University of Petroșani

This investigation is an effect-type investigation, because students are the receptors or the beneficiary of the online education system, so it was important to determine how they react to the large variety of communication and teaching methods used by teachers for implementing an online education system. Even though the University of Petrosani is a small university, the methods used by teachers in the online teaching environment were very diverse, caused by the diverse disciplines from the curricula, so the effects are also diverse.

Using a five-point scale for the level of hardware tools used for participating to online classes (5 = very used, 4 = used, 3 = medium used, 2 = less used, and 1 = not used), it is relevant to remark that students are especially using smart phones and laptops for attending online classes (Table 21).

Using again the five-point scale for the level of communication methods used for participating to online classes (5 = very used, 4 = used, 3 = medium used, 2 = less used, and 1 = not used), it is relevant to remark that students are especially using email and Zoom video conference software for communicating in the online teaching environment (Table 22).

In Table 23, the authors had analyzed the correlation between independent variable, the attendance level in online classes measured in hours per day, with the dependent variable, the attendance level in specialized type of online classes such as meetings and virtual classes. Pearson product moment correlation analysis was used to determine the nature, direct or inverse, and the degree of association between variables, while multiple regions were used to determine the explanatory power of independent variables over the dependent variable. The correlation matrix shows the strength of the association between the variables and is presented above in Table 23. The table above shows the cross-correlation coefficients of the main constructs in this study. As the table indicates, the specialized online tools, such as meeting, and especially virtual classes, are significant and positively related to adequate online teaching with the correlation coefficient (*p* < 0.01). Therefore, the authors conclude that there is a strong, significant, and positive association between these two constructions.

In Table 24, the authors had analyzed a correlation between independent variable, the number of teaching methods used in online classes, with the dependent variable, the number of software used, communication methods or testing methods. Pearson product moment correlation analysis was used to determine the nature, direct or inverse, and the degree of association between variables, while multiple regions were used to determine the explanatory power of independent variables over the dependent variable. The correlation matrix shows the strength of the association between the variables and is presented above in Table 24. The table above shows the cross-correlation coefficients of the main constructs in this study. As the table indicates, the specialized software tools used for teaching, communicating, and testing are positively related to adequate methods of teaching, with the correlation coefficient (*p* < 0.01); however, based on the small values of moment correlations, the authors conclude that there is a not a strong and significant association between these two constructions.

The standard deviation from Table 18, Table 19, Table 20, Table 21 and Table 22 shows that data are not dispersed, so data extension is low, and the results are useful in establishing the representativeness of the centrality measures and is relevant in estimating statistical parameters and statistical prediction. Pearson r moment correlation, presented in Table 23 and Table 24, shows a strong correlation for the variable presented in Table 23, and a poor correlation for the variable presented in Table 24.

### 4.3. Statistical Analyzes Results

In the statistical analysis performed by the authors, they demonstrated that the three different surveys, conducted in January–February 2021, validated the assumption that there is a strong relationship between the online teaching effort of the professors in two different levels of education, such as academic and primary and secondary, and the effect on the students and the evolution of some online methods over two different academic years. They conducted a paired samples t-test in order to demonstrate the effect that the implementation of the online education in the Romanian education system would have over the students.

#### 4.3.1. Benchmark 1—Academic Teachers’ Point of View versus Primary and Secondary Teachers’ Point of View via the Testing Method Used: Online Testing Methods Are Applied in the Same Way by Professors from Different Levels of Education

It is found that the t-test is not significant because the significance level (*p*-value) is 0.076, so greater than 0.05. In this case, the repeated-measures t-test demonstrates that the null hypothesis H1 is not rejected. This suggests that effects of online testing methods applied by academic teachers do not affect online testing methods applied by primary and secondary education system teachers, so our hypothesis is not supported. Because the null hypothesis is not rejected, it can be considered that the observed differences are due to chance, and the result is statistically insignificant (Table 25).

#### 4.3.2. Benchmark 2—Teachers’ Point of View versus Students’ Point of View for the University of Petroșani via the Teaching Method Used: Online Teaching Methods are Perfectly Received by Students from Professor in the Academic Level of Education

It is found that the t-test is significant because the significance level (*p*-value) is 0.035, <0.05, and in this case, the t-test is significant for 96.5% of cases. A repeated-measures t-test found this difference to be significant, and values t (5) = 2.882 and *p* = 0.03 suggest that online teaching method perceptions of students may be affected by online teaching methods applied by academic teachers, and this result is supporting our hypothesis, because the decision rule when *p* < 0.05 is to reject the H2 hypothesis, if the computed test statistic is less than −1.96 or more than 1.96 (t = 2.882 > 1.96), in favor of our experimental hypothesis. By rejecting the null hypothesis, the authors state that the observed results are not due to chance, so the result obtained is statistically significant (Table 26).

#### 4.3.3. Benchmark 3—Students in the 2019/2020 Academic Year versus Students in the 2020/2021 Academic Year for the University of Petroșani: The Most Preferred Hardware Equipment Used by Students in Online Education Remains the Same in Two Different Academic Years

It is found that the t-test is not significant, as the significance level (*p*-value) is 1.000, so greater than 0.05. In this case, the repeated-measures t-test demonstrates that the null hypothesis H3 is not rejected. This suggests that effects of the devices used for online education by students in 2020 do not affect the use of devices for 2021, so our hypothesis is not supported. Because the null hypothesis is not rejected, it can be considered that the observed differences are due to chance and the result is statistically insignificant (Table 27).

#### 4.3.4. Benchmark 4—Summer Exam Session 2020 versus Winter Exam Session 2021 for the University of Petroșani, so H3 is Accepted: The Testing Methods Applied on Students in Online Education Remains the Same in Two Different Academic Years

It is found that the t-test is significant, as the significance level (*p*-value) is 0.049, so less than 0.05, and in this case, the t-test is significant for 95.1%. A repeated-measures t-test found this difference to be significant, and the values t (5) = 2.587 and *p* = 0.049 suggest that online testing methods for two different years may be affected by online teaching methods applied by academic teachers, and this result is supporting our hypothesis, because the decision rule when *p* < 0.05 is to reject the H4 hypothesis, if the computed test statistic is less than −1.96 or more than 1.96 (2.587 > 1.96), in favor of our experimental hypothesis. The authors reject the null hypothesis in favor of the experimental hypothesis (Table 28).

In conclusion, hypotheses H2 and H4 are validated, and hypotheses H1 and H3 cannot be demonstrated.

Obviously, the study tried to investigate the education before the COVID-19 pandemic in the classic method of education, and the situation during the pandemic when online methods were used. The RPSES and small Romanian universities have not used online education before the pandemic, and the e-learning education methods only at an unimportant level. In Romania, directing education from class via distance was the most used method during the pandemic, and the result over the three education stakeholders analyzed can be summarized in a short phrase. Online education represents, for students a second nature, and teachers are now, after one year of online education, more prepared and trained, and also the transition to distance learning is now easier because different software companies have created resources that could help both teachers and students to migrate more easily to the online system. Another obvious conclusion is that Romanian education managers did not have an honorable reaction to this change, and the teachers were forced to do the same activities as before the pandemic.

## 5. Discussion

As it can be seen in the Literature Review section, the COVID-19 pandemic has led researchers around the world to investigate how education has been affected by the online system.

The authors analyzed the main methods used to implement online education. The three different studies allowed the authors to provide comparative analysis and also a statistical analysis. Through these methods, they studied the essential tools used in online education, such as teaching and meeting tools, homework and project collaboration methods, testing methods, and, of course, virtual classrooms, which include all the first three tools. The authors also studied the hardware devices used for online education and the communication methods.

Another aspect analyzed by the authors was to estimate COVID-19’s impact on the Romanian education system. This impact was determined primarily by considering the number of students or pupils participating in online education during COVID-19 compared to previous participation before COVID-19, but also by using questions based on Likert scale for participants’ satisfaction. In fact, the purpose of the study was to find an answer to three important questions, such as: what the perception of students was and how were they affected by online education, how teachers adapted to the new education system, and how managers from the education system effectively manage distance education.

Although this study has some limitations in the academic education investigation, these do not invalidate the results obtained [52]. Even if the study reveals the points of view of a large population of professors from the Romanian primary and secondary education system, and also the majority of professors and a quarter of the students from a small Romanian university, the results are limited in terms of their level of generalizability because of cultural contexts and participants’ particularities from each region. Another limitation is related to the item construction that measured the level of online education implementation, but also the future research design could be improved. Self-reporting and self-evaluation can also be a risk factor because of social desirability. Other limitations are caused by not using random sampling, and also by not including students from the RPSES system in the investigations. The authors investigated only one university, the University of Petrosani, a small university that could be considered representative not for the whole Romanian academic education system, but only for the small Romanian universities. This assumption can be based on the fact that primary, secondary, and high schools, and small universities also, are similar in fields of online education, because, unfortunately, they started the massive implementation and use of e-learning only after 15 March 2020.

The investigation could also be interpreted from the Romanian PISA results perspective by regarding the item school segregation, and gaps in material and staff shortage between advantaged and disadvantaged schools. The authors have found from this perspective that in Romania, low-performing students are clustered in certain schools to the same extent as the Organization for Economic Cooperation and Development (OECD) average, and high-performing students more often clustered, and also many students, especially disadvantaged students, hold lower ambitions than would be expected given their academic achievement [53,54,55,56,57,58]. In Romania, about one in four high-achieving disadvantaged students—but only about 1 in 30 high-achieving advantaged students—do not expect to complete tertiary education. Correlated with the pandemic situation and the forced online education over this period, Romania will be probably one of the few EU member states with major problems regarding the influence of poverty on a possible hybrid educational system.

## 6. Conclusions

The analysis of the online Romanian education system after one year of COVID-19 could be also seen as a social phenomenon, because the Romanian education system affected many elements of Romanian society through the pupils’ or students’ parents. Another involved part of the society was obviously the management of the education system, which was not prepared for e-learning or online education at the beginning of the pandemic crisis, and also responded inefficiently to this challenge.

To be able to answer to the most important question of the study—the effect of online education system over the students from the Romanian education system from different perspectives—the authors conducted a relevant study in the Romanian education system in the age of COVID-19.

The study was based on three surveys made in the year 2021, and also one survey made in the year 2020; this last one used in this paper only for two comparative analyses. The authors had also conducted comparative analysis, the first of them between the perception of online education of the primary and secondary education teachers and the perception of academic teachers, and the second one regarding the perspective on the online education from the teachers’ and students’ opinion, and the last two as comparisons of the situation of online education in the University of Petroșani at the end of the academic year 2019/2020 and the situation at the end of the first semester of the academic year 2020/2021.

The lack of hardware and software infrastructure for these 13–19% of Romanian pupils is validated by the data sent by the Romanian Ministry of Education to the Ministry of European Investments and Projects, where it is reported that a number of 391,726 people fall within the criteria of Government Emergency Ordinance no. 133/2020 to obtain educational support. At the level of the 2019–2020 school year, the Ministry of Education and Research has identified a total number of 302,173 children who fall into the categories of the target group related to the measure regulated by this normative act dedicated to disadvantaged students who receive educational support.

The answer to the first study objective was found in the first two investigations. The first investigation confirmed, by analyzing the collected data, that 13% to 19% of the pupils and teachers from the primary and secondary Romanian education system did not take part in online classes for various reasons, but probably because of the lack of hardware and software infrastructure. On the other hand, the authors conclude that 49% of the academic teachers from investigation 2 declare that online education represents a progress for the educational system, because this process reached a 50% increase in the number of participants in the online classes. So, in the authors’ opinion, Romanian teachers coped well with the challenge of online education.

The answer to the second study objective can be stated in the conclusion that most of the schools from the Romanian education system have migrated quickly to the online education system. Therefore, the study reveals that over 82% of the students have participated rhythmically in online education. In addition, in the second investigation, the authors demonstrated that the drop of 8% in the participation of the students in online classes is caused by their teachers’ deficiencies. The authors demonstrated that 16% of the students participate only in 50% of the online classes, and this represents one, two, or three courses out of seven, because of technical difficulties, because of a reduced involvement of the teachers or by a low level of IT skills. In addition, considering that the highest level of online teaching is represented by the use of e-learning platforms, and in fact, by the use of virtual classes, meeting solutions, and online quizzes, the authors demonstrated that 38% of the students participate in only 50% of the online classes conducted via a virtual class, probably because of the lack of training of some teachers assigned to the other 50% of disciplines from the curricula.

The final result of the paper is the analysis of how the future of education in Romania after the COVID-19 age will look, considering that the future education system will include the online methods as sustainable assessment, tested and improved in this period. Therefore, after the challenge that COVID-19 has presented for the education system, a challenge to which students and pupils took the pole position and responded very well, probably because students are native digitals, the result is that this COVID-19 online education age must be used in the future by education managers. Education managers are the third part of this educational triad, besides students and teachers, and they answered unsatisfactorily to this challenge. The study demonstrates that one of the most unprepared actors from the education system were the mangers from the education. Even if the real challenge was for the teachers and their students, these actors managed, relatively well, the online education, but some of the education managers were a disappointment for the Romanian society. This could be considered the answer to the third study question, because the future is always influenced by the management level of a system.

Developing virtual courses for the new generations will present a great challenge for worldwide education systems from now on. Twenty years ago, Marc Prensky coined the term “digital native”, now commonly applied to both Millennials and Gen Zers. The term implied that this new generation of students was emerging and that educators would need to adapt to their technology preferences and learning styles. The challenges of engaging them in an impactful way in a new education system have been further exacerbated by the COVID-19 pandemic, which has necessitated a rapid and abrupt shift to a virtual learning model.

The realities of this period have given the undeniable conclusion that Romanian mangers from the education system inefficiently managed the COVID-19 crisis, and in the future they must improve their contribution to the educational act, by including the online methods tested and improved during this period as a sustainable evaluation in the future educational system, in the curricula, and in the teaching methods, because, probably, the future education will become a hybrid one.

Considering that, probably, education will become, in future, more and more a hybrid one, the authors set out, as a future objective, to develop the present research for the whole Romanian education system and for all the stakeholders involved. The authors will also continue their effort regarding raising the awareness of the leadership of the Romanian Ministry of Education every year in this field of hybrid education.

## Figures and Tables

**Table 1:** Summary of processed cases of variables: reliability statistics.

Variable	Cronbach’s Alpha
Online course in Academic Education	0.850
Communication methods in Academic Education	0.820
Teaching methods in Academic Education	0.758
Online course in Romanian Primary and Secondary Education	0.771

**Table 2:** The number of respondent teachers compared with the total number of teachers from the Romanian education system [50].

Regions in Romania	Number of Respondents	Total Number of Romanian Teachers
Northwest	122	35,735
Center	669	27,086
Southeast	768	24,378
Bucuresti	340	20,301
South Muntenia	379	27,963
West	244	18,184

**Table 3:** The academic level where the respondent teachers are included (question 2).

International Standard Classification of Education (ISCED) Level	Respondents
ISCED 0: Early childhood education	389
ISCED 1: Primary education	589
ISCED 2: Lower secondary education	919
ISCED 3: Upper secondary education	621

**Table 4:** The number of students who answered the questionnaire from the Mining and Science Faculties of the University of Petroșani.

Faculty	Level of Study	Average Age of Students	Number of Students
Mining	Bachelor’s	32	174
Science	Bachelor’s	26	111
Mining + Science	Bachelor’s	29	285
Mining	Master’s	37	107
Science	Master’s	35	133
Mining + Science	Master’s	35	240
Mining + Science	Bachelor’s + Master’s	32	525

**Table 5:** The average number of students participating in the class before the beginning of the pandemic and the average number of students participating in the class during the pandemic (question 3 and question 4).

Level of Education	Average Number of Students in Class before COVID-19	Average Number of Students in Class during COVID-19	Attendance during COVID-19 (%)	Decrease (%)
Primary	19.03	15.57	82%	18%
Secondary	25.57	22.17	87%	13%
Total	23.5	19.63	85%	15%

**Table 6:** The percentage of which teachers managed to teach online courses during COVID-19 compared to the classic version, prior to COVID-19 (question 2).

Percentage of Which Teachers Managed to Teach Online Courses during COVID-19 Compared to the Classic System	Number of Teachers	Percentage Out of 2540 Respondents
Identical	948	37%
Slightly reduced from 80% to 99%	1107	44%
Reduced from 50% to 79%	390	15%
Very low below 50%	95	4%

**Table 7:** Percentage of which teachers managed to teach online courses during COVID-19 compared to the classic system, prior to COVID-19 (question 1).

Percentage of Which Teachers Managed to Teach Online Courses during Pandemic Compared to the Classic System	Number of Teachers Answers	Percentage out of 80 Respondents
Identical	41	51
Slightly reduced from 80% to 99%	29	36
Reduced from 50% to 79%	9	11
Very low below 50%	1	1

**Table 8:** Participation of students in online courses compared to the participation in classes conducted in the classic version, prior to the pandemic, in the teacher’s opinion (question 2).

Percentage of Students during Pandemic Compared to the Classic System	Number of Teachers Answers	Percentage out of 80 Respondents
Higher, from 110% to 150%	39	49
Identical	13	16
Slightly reduced from 80% to 99%	15	19
Reduced from 50% to 79%	12	15
Very low below 50%	1	1

**Table 9:** The percentage of which students managed to take part in courses during the pandemic compared to the participation in the classic version, in the pre-pandemic (question 4).

Percentage of Students that Managed to Take Part in Online Courses during Pandemic Compared to the Classic System	Number of Students	Percentage out of 551 Respondents
Identical	202	37
Slightly reduced from 80% to 99%	183	33
Reduced from 50% to 79%	96	17
Very low below 50%	70	13

**Table 10:** Teaching method used during the COVID 19 pandemic (question 8).

Respondent/Teaching Method	Total	Primary	Secondary
PowerPoint presentation	65%	65%	66%
Audio video presentations, video conference	79%	83%	77%
Lecture (without visual materials)	22%	19%	24%
Collaborative projects on small groups	18%	11%	23%
E Content (electronic content related to e-learning platforms)	21%	16%	25%
Other	37%	39%	36%

**Table 11:** Video conferencing or meeting software used for teaching during the COVID-19 pandemic (question 6).

Respondents/Video Conferencing Application	Total	Primary	Secondary
Zoom	33%	34%	32%
Google Meet	72%	62%	79%
Microsoft Teams	10%	6%	13%
Microsoft Skype	1%	0%	1%
WhatsApp	32%	37%	29%
Other	7%	8%	6%
None	1%	1%	0%

**Table 12:** Collaborative platform or virtual classroom used for student management, transmission of materials, homework, or progress evaluation during the COVID 19 pandemic (question 7).

Respondents/Collaborative Platform or Virtual Classroom	Total	Primary	Secondary
Google Classroom	75%	67%	81%
Microsoft OneNote	2%	1%	3%
Class Marker	0%	0%	0%
Own specialized platform	3%	3%	3%
Edmodo	1%	1%	1%
ClassDojo	0%	1%	0%
Easy Class	0%	0%	0%
Other	23%	28%	20%

**Table 13:** Testing or examination method used during the COVID 19 pandemic (question 9).

Respondents/Testing or Examination Method	Total	Primary	Secondary
Google Forms	42%	23%	54%
Microsoft Forms	5%	2%	6%
Class Marker	2%	1%	3%
Own specialized platform	7%	7%	7%
Other	58%	62%	56%

**Table 14:** Homework transmission method used during the COVID 19 pandemic (question 10).

Respondents/Homework Transmission Method	Total	Primary	Secondary
Virtual classroom (Google Classroom…)	79%	68%	87%
Email	11%	5%	14%
WhatsApp	50%	56%	46%
Other	16%	18%	14%

**Table 15:** Comparison between median number of classes held during COVID-19 and prior.

Faculty	Level	Median Number of Hours during COVID-19/Day Online	Median Number of Hours Prior COVID-19/Day Classical	Influence of COVID-19 %
Mining	Bachelor’s	3.80	3.61	105%
Science	Bachelor’s	4.17	4.51	92%
Mining + Science	Bachelor’s	3.93	3.95	100%
Mining	Master’s	2.59	3.08	84%
Science	Master’s	2.52	3.33	76%
Mining + Science	Master’s	2.56	3.20	80%

**Table 16:** Out of the total of 5–7 study disciplines allocated to the first semester of the 2020–2021 academic year, how many of them did you take online courses? (question 13); how many of them have been conducted by teacher through a video conferencing system? (question 14); how many of them have been conducted by teacher through an e-learning platform (virtual classroom or online testing)? (question 15).

Courses out of 7	Number of Students Participant to Online Conducted Courses out of 7 from the Curricula	% of Courses Conducted Online	Number of Students Participant to Video Conference Conducted Courses out of 7 from the Curricula	% of Courses Conducted Online	Number of Students Participant to e-Learning Conducted Courses out of 7 from the Curricula	% of Courses Conducted Online
0	0	0%	0	0%	0	0%
1	5	1%	20	4%	100	18%
2	13	2%	22	4%	49	9%
3	25	5%	46	8%	60	11%
4	62	11%	71	13%	55	10%
5	177	32%	166	30%	131	24%
6	84	15%	84	15%	54	10%
7	185	34%	142	26%	102	19%

**Table 17:** Summer exam session 2020 versus winter exam session 2021 [51].

Year	2020	2021	2020	2021
Faculty/Exam	Faculty of Mining	Faculty of Mining	Faculty of Science	Faculty of Science
Homework Handouts	40%	30%	33%	16%
Video Conference Exam (WhatsApp)	12%	0%	6%	1%
Quizzes or Forms	52%	28%	45%	58%
Video Conference Exam	48%	7%	16%	16%
Video Conference Exam + Quizzes	19%	35%	2%	9%

**Table 18:** Descriptive statistics of the number of students attending classes before and during COVID-19.

Variable	*N*	Minimum	Maximum	Mean	Standard Deviation
No_Students_before_COVID	2540	1	99	21.34	8.418
No_Students_during_COVID	2540	0	99	18.11	8.567
Valid *N*	2540				

**Table 19:** Descriptive Statistics for main parameters of the online system.

Variable	*N*	Minimum	Maximum	Mean	Standard Deviation
No_of_Teaching_methods	2540	1	6	2.42	1.147
No_of_Videconference_softwares	2540	0	5	1.54	0.786
No_of_Virtual_classrooms	2540	0	3	1.04	0.327
No_of_Homework_transmision_methods	2540	1	4	1.55	0.684
No_of_Testing_methods	2540	0	4	1.14	0.577
Valid *N*	2540				

**Table 20:** Descriptive statistics for the teaching level and the attendance level.

Variable	*N*	Minimum	Maximum	Mean	Standard Deviation
Level_of_teaching	80	1	4	3.38	0.736
Level_of_attendence	80	0	5	3.91	1.265
Valid *N*	80				

**Table 21:** Descriptive statistics of hardware tools used for online courses.

Variable	*N*	Minimum	Maximum	Mean	Standard Deviation
Desktop	551	1	5	2.32	1.524
Laptop	551	1	5	3.66	1.419
Smart Phone	551	1	5	3.90	1.227
Tablet	551	1	5	1.39	0.964
Valid *N*	551				

**Table 22:** Descriptive statistics of the communication method used for online courses.

Variable	*N*	Minimum	Maximum	Mean	Standard Deviation
email	551	1	5	4.74	0.576
WhatsApp	551	1	5	3.00	1.433
Phone	551	1	5	2.57	1.433
Facebook	551	1	5	2.36	1.356
Google	551	1	5	1.88	1.206
Teams	551	1	5	1.83	1.217
Zoom	551	1	5	4.58	0.872
Valid *N*	551				

**Table 23:** Correlations matrix of major variables related to the online level.

Variable	No_Online_Courses	No_Online_Videoconference_Courses	No_e-learning_Platform_Courses
No_online_courses	1		
No_online_videoconference_courses	0.676 *	1	
No_e-learning_platform_courses	0.427 *	0.507 *	1

* Correlation is significant at the 0.01 level (2-tailed). *N* = 551.

**Table 24:** Correlations matrix of major variables for main parameters of the online teaching method.

Variable	No_of_Teaching_Methods	No_of_Videoconference_Soft	No_of_Project_Transmision_Methods	No_of_Testing_Methods
No_of_Teaching_methods	1			
No_of_Videoconference_software	0.214 *	1		
No_of_Project_transmision_methods	0.344 *	0.253 *	1	
No_of_Testing_methods	0.196 *	0.233 *	0.210 *	1

* Correlation is significant at the 0.01 level (2-tailed). *N* = 551.

**Table 25:** Paired samples t-test applied for the perspective of primary and secondary education level teachers and perspective of academic teachers on online testing methods (Quizzes and Forms).

Paired Samples Correlations					Paired Samples Test									
							Paired Differences					t	df	Sig. (2-tailed)
*N*	Correlation	Sig.					Mean	Standard Deviation	Standard Error Mean	95% Confidence Interval of the Difference				
										Lower	Upper			
Pair 1	Teachers_Primary_Secondary & Teachers_University	6	0.975	0.001	Pair 1	Teachers_Primary_Secondary-Teachers_University	874.500	959.686	391.790	−132.628	1881.628	2.232	5	0.076

**Table 26:** Paired samples t-test applied for the perspective of teachers and perspective of students on online teaching methods.

Paired Samples Correlations					Paired Samples Test									
							Paired Differences					t	df	Sig. (2-tailed)
		N	Correlation	Sig.			Mean	Standard Deviation	Standard Error Mean	95% Confidence Interval of the Difference				
										Lower	Upper			
Pair 1	No_students & No_teachers	6	0.942	0.005	Pair 1	No_students-No_teachers	157.833	134.141	54.763	17.061	298.606	2.882	5	0.035

**Table 27:** Paired samples t-test applied for the perspective of students in two different academic years on most preferred hardware equipment for online classes.

Paired Samples Correlations					Paired Samples Test									
							Paired Differences					t	df	Sig. (2-tailed)
		N	Correlation	Sig.			Mean	Standard Deviation	Standard Error Mean	95% Confidence Interval of the Difference				
										Lower	Upper			
Pair 1	Equipment_very_used_2021 & Equipment_very_used_2020	4	0.998	0.002	Pair 1	Equipment_very_used_2021 - Equipment_very_used_2020	0.00000	0.04082	0.02041	−0.06496	0.06496	0.0	3	1.0

**Table 28:** Paired samples t-test applied for the perspective of students in two different academic years exam sessions on different methods of conducting an exam.

Paired Samples Correlations					Paired Samples Test									
							Paired Differences					t	df	Sig. (2-tailed)
		N	Correlation	Sig.			Mean	Standard Deviation	Standard Error Mean	95% Confidence Interval of the Difference				
										Lower	Upper			
Pair 1	University_2020 & University_2021	6	0.961	0.002	Pair 1	University_2020-University_2021	41.167	38.984	15.915	0.255	82.078	2.587	5	0.049

## Data Availability

The data presented in this study are available on request from the first author.
